# Electrodeposition of Nanostructured Metals on n-Silicon and Insights into Rhodium Deposition

**DOI:** 10.3390/nano14242042

**Published:** 2024-12-20

**Authors:** Giulio Pappaianni, Francesco Montanari, Marco Bonechi, Giovanni Zangari, Walter Giurlani, Massimo Innocenti

**Affiliations:** 1 Dipartimento di Chimica “Ugo Schiff”, Università degli Studi di Firenze, Via della Lastruccia 3, 50019 Sesto Fiorentino, Italy; 2 Consorzio Interuniversitario Nazionale per la Scienza e Tecnologia dei Materiali (INSTM), Via G. Giusti 9, 50121 Florence, Italy; 3 Department of Materials Science and Engineering, University of Virginia, Charlottesville, VA 22904, USA

**Keywords:** electrodeposition, silicon, rhodium, SEM, XPS, semiconductors

## Abstract

In this study, we investigate the electrodeposition of various metals on silicon. Mn, Co, Ni, Ru, Pd, Rh, and Pt were identified as promising candidates for controlled electrodeposition onto silicon. Electrochemical evaluations employing cyclic voltammetry, Scanning Electron Microscopy (SEM) associated with energy-dispersive X-Ray Spectroscopy (SEM-EDS), and X-Ray Photoelectron Spectroscopy (XPS) techniques confirmed the deposition of Pd, Rh, and Pt as nanoparticles. Multi-cycle charge-controlled depositions were subsequently performed to evaluate the possibility of achieving tunable electrodeposition of nanostructured rhodium on n-doped silicon. The procedure increased surface coverage from 9% to 84%, with the average particle size diameter ranging from 57 nm to 168 nm, and with an equivalent thickness of the deposits up to 43.9 nm, varying the number of charge-controlled deposition cycles. The electrodeposition of rhodium on silicon presents numerous opportunities across various scientific and technological domains, driving innovation and enhancing the performance of devices and materials used in catalysis, electronics, solar cells, fuel cells, and sensing.

## 1. Introduction

The electrodeposition of metal films, patterns, and nanostructured materials on silicon substrates provides a means to combine the desirable properties of metals with the exceptional electronic properties of silicon, providing useful tools in the fields of integrated circuits, electrodes in batteries, supercapacitors, fuel cells, sensors, water-splitting devices, and photovoltaic cells [1,2,3].

Currently, thin film depositions on silicon are obtained mainly using vapor deposition methods. Among physical deposition methods are physical vapor deposition (PVD) [4], including vacuum evaporation [5], molecular beam epitaxy (MBE) [6], sputtering [7], and pulsed laser deposition (PLD) [8]. Other physical techniques for thin film synthesis include spin coating [9] and spray coating [10].

On the other hand, the electrodeposition of thin films and metal nanoparticles onto semiconductors offers numerous advantages and presents high technological importance, especially for metal/semiconductor contacts. This deposition technique is an inexpensive, easily scalable, and quick synthesis route that shows low costs, is carried out at room temperature and atmospheric pressure, in aqueous solution, and with adjustable deposit properties [11,12].

Electrochemical Atomic Layer Deposition (E-ALD) allows the deposition of variable-thickness thin films on an electrode substrate, controlling the morphology and composition of the deposited film with a low percentage of defects and contaminations [13]. This technique also enables the deposition of atomic monolayers by utilizing surface-limited reactions (SLRs), such as underpotential deposition (UPD) [14,15,16]. Furthermore, E-ALD is often limited by the need to use substrates with high crystalline order, exhibiting a strong affinity for deposition and allowing morphologically ordered growth. As a result, this technique has primarily focused on the use of crystalline gold [17] and silver substrates [18]. However, due to the high cost of these materials, their widespread industrial use for thin film production is currently challenging. Instead, the use of monocrystalline silicon wafers represents a viable alternative as substrates, thanks to the relatively low cost and the large availability due to the abundance of Si in the Earth’s crust and the large production for the electronics sector.

Despite UPD offering greater quantitative control over the deposition process, electroless deposition has garnered significant interest due to its simplicity. The latter technique is particularly studied for metals like copper (Cu), gold (Au), and platinum (Pt) due to their excellent conductivity and chemical stability in technological contexts, such as microelectronics [19]. The field of electrodeposition of metals on silicon still presents great room for improvement in semiconductor–metal contact technology [20,21], and it shows technological relevance, as it leads to the formation of the Schottky barrier [22]. This barrier allows conduction in one polarity but not in the opposite, making it crucial for various applications in electronics. When a semiconductor comes into contact with an electrolyte during the deposition process, the resulting barrier is known as the Mott–Schottky junction [23]. The deposition of a continuous ultrathin film (<50 nm) onto silicon is not straightforward due to the band structure of the semiconductor, which affects both the thermodynamics and the kinetics of metal deposition processes [24,25,26]. Most of the literature reports on the deposition of rather thick and clustered films. An excellent example of an ultra-thin film is reported by Allongue with the production of a continuous epitaxial gold film of just 4 nm under the mass transport limit [27]. However, there is still no evidence of works in which an SLR is reported.

In this paper, the deposition of technologically relevant metals on silicon substrates through electrodeposition was investigated, exploring the possibility of obtaining monolayers, thin films, and nanostructured deposits. A metal monolayer can serve as a platform for further surface functionalization, such as introducing functional groups, nanoparticles, or molecular species onto the surface. This could open up possibilities for applications in molecular electronics, biosensors, and surface-enhanced spectroscopies.

The first selection of metals to be electroplated was made through the evaluation of Density Functional Theory (DFT) calculations, considering the metal–silicon formation energy, allowing the evaluation of which metals had a higher affinity with silicon compared to the metal itself [28].

Voltammetric studies were carried out using solutions containing the metals Mn, Co, Ni, Ru, Pd, Rh, and Pt to investigate the presence of cathodic processes related to metal deposition processes. The possibility of obtaining atomic monolayers or ultra-thin deposits was evaluated. The risk of uncontrolled electroless depositions was also assessed. Finally, controlled charge electrodeposition measurements were performed on the most promising metal. Rhodium showed the best results; therefore, multi-cycle charge-controlled depositions were performed to evaluate the possibility of obtaining a tunable nanostructured deposit and/or a thin film. To the best of our knowledge, there are limited studies specifically addressing the electrodeposition of nanostructured rhodium on silicon substrates [29]. Electrodeposition of nanostructured rhodium metal on silicon and the ability to achieve different surface coverings as operating conditions change is an important result for many high-impact technology applications, especially in hydrogen and oxygen evolution reactions, thanks to the excellent catalytic properties of rhodium [30,31]. Moreover, rhodium-coated silicon can be used to develop sensitive and selective sensors [32], robust interconnects and contacts in semiconductor devices, and to fabricate nanoscale electronic devices [33], to improve the efficiency of silicon-based solar cells [34], in the development of LEDs, where the rhodium layer can enhance the emission efficiency [35], and in corrosion resistance and protective coatings that extend the lifespan and reliability of electronic and mechanical components [36]. The results obtained in this work show potential applications in the cutting-edge fields of photonic devices for scalable quantum information applications [37], enhanced electrocatalytic oxidations in direct alcohol fuel cells [38], and photoinduced wave modulation using hybrid metal–silicon metasurfaces [39].

## 2. Materials and Methods

### 2.1. Reagents

All the solutions were prepared using ultrapure water (<0.059 µS/cm). The following reagents were used for the RCA (Radio Corporation of America) procedure [40,41] for the activation of the silicon electrode: sulfuric acid H_2_SO_4_ (98%) supplied by chemPUR, Karlsruhe, Germany; hydrochloric acid HCl (37% *w*/*w*) and hydrofluoric acid HF (48% *w*/*w*) from VWR International, Fontenay-sous-Bois, France; acetone AcOH, ethanol EtOH, and hydrogen peroxide H_2_O_2_ (30% *w*/*w*) supplied by Carlo Erba, Val de Reuil, France. The following salts were used for the electrolytic solutions: rhodium (III) chloride hydrate RhCl_3_·xH_2_O, supplied by chemPUR, Karlsruhe, Germany; potassium (II) tetrachloroplatinate K_2_PtCl_4_, and cobalt (II) sulfate heptahydrate CoSO_4_·7H_2_O, by Sigma-Aldrich, St. Louis, MO, USA; manganese (II) sulfate monohydrate MnSO_4_·H_2_O, from Merck KGaA, Darmstadt, Germany; Hexaammineruthenium (III) chloride [Ru(NH_3_)_6_]Cl_3_, supplied by Acros Organics, Geel, Belgium; tetraammine palladium (II) sulfate, [Pd(NH_3_)_4_]SO_4_, by Thermo Fisher Scientific, Waltham, MA, USA; nickel (II) sulfate hexahydrate NiSO_4_·6H_2_O from Carlo Erba, Val de Reuil, France. For the studies conducted in this work, metal solutions with a concentration of 1 mM metal salt in 0.1 M sulfuric acid were used. Furthermore, a 0.1 M sulfuric acid solution was prepared for instrument treatment and reference measurements.

### 2.2. Experimental Setup

The experimental setup used in this work for the electrochemical characterization and deposition is a fully computerized system that allows control over the solution input and permanence time in the cell using a solenoid valve system [16,42]. The solutions are stored in Pyrex glass, and deaerated with nitrogen gas, and are remotely injected in a Kel-F (polychlorotrifluoroethylene) electrochemical cell.

The potentiostat used is an AMEL, Model 551, and the personal computer is equipped with a National Instruments data acquisition card. A three-electrode setup was used: a monocrystalline (100) n-Si (0.1–1 Ω·cm) working electrode, with an exposed electrode surface of 0.785 cm^2^; a gold counter electrode; and an Ag/AgCl saturated KCl reference electrode. All cyclic voltammetry measurements were conducted at a scan rate of 10 mV/s.

Before the measurements, the silicon electrodes were cleaned and activated using the RCA reported in Table S1.

SEM analyses were performed using a Hitachi SU3800 (Hitachi High-Tech Corporation, Tokyo, Japan) that was equipped with an Ultim Max 40 Analytical Silicon Drift EDS Detector (Oxford Instruments plc, Abingdon, UK). EDS measurements were carried out using the Layer Probe program of AZteclive (Version 5.0) to investigate the average equivalent thickness of the metallic rhodium deposit.

XPS was performed using an instrument equipped with a non-monochromatic X-ray source (VSW Scientific Instrument Limited model TA10, Al Kα radiation 1487.7 eV, set to work at 120 W (12 kV and 10 mA), and a hemispherical analyzer (VSW Scientific Instrument Limited model HA100, Manchester, UK). The analyzer was equipped with a 16-channel detector and a dedicated differential pumping system maintaining the pressure in the chamber below the 10−8 mbar range. The pass energy was set to 22 eV. The measured spectra were analyzed using CasaXPS software (version 2.3.19, Casa Software Ltd., Teignmouth, UK). The inelastic background was subtracted using Shirley’s method [43], and mixed Gaussian (70%) and Lorentzian (30%) contributions were used for each component. Calibration of the spectra was obtained by imposing the lowest component relative to the 1 s transition of carbon for adventitious carbon at 284.8 eV and the signal of elemental silicon at 99.4 eV [44,45]. For the fitting of each peak of the three elements analyzed, the Handbook of Photoelectron Spectroscopy was consulted [46].

## 3. Results and Discussion

### 3.1. Metals’ Selection

The initial selection of metals for the electrodeposition on the silicon substrate was conducted through the study of the data obtained from computational calculations thanks to The Open Quantum Materials Database (OQMD) [47,48,49] based on Density Functional Theory (DFT) at the PBE/PAW level. The data were also compared with those of the Formation Energy Predictor [50,51,52,53] data mining algorithms of Northwestern University (Evanston, IL, USA) using PRB’14, ICDM’16, and ElemNet models and Material Project [54] DFT calculations at the PBE/PAW level. The data obtained from these alternatives is mostly in line with OQMD results.

**Figure 1:**
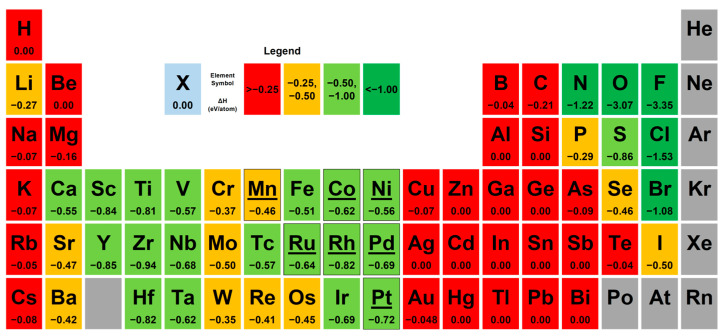
Periodic table of elements highlighting the formation energy (eV/atom) with silicon; the metals used in this work are highlighted (nickel, ruthenium, palladium, rhodium, platinum, manganese, and cobalt).

Figure 1 reports the formation energies (eV/atom) of each element with silicon, considering the most stable phase. This study focused on identifying elements with favorable formation energies for bonding with silicon.

The feasibility of electrodeposition of the elements was also evaluated [55], as well as observing the theoretical value of the metal–silicon Schottky barrier, which is a factor to consider for device applications [21,56]. For instance, in an ohmic contact, a very low Schottky barrier is preferable to obtain a linear current with no potential barrier in either direction; in fact, the lower the barrier height, the lower the specific contact resistivity is [57]. After considering the above, the following metals were selected: nickel, ruthenium, palladium, rhodium, platinum, manganese, and cobalt.

The possibility of achieving electroless deposition for all the metals used in this paper was also evaluated. If a metal undergoes electroless deposition under these experimental conditions, that metal is not ideal for achieving controlled deposition. Details are given in Supplementary Materials. Only the Pt-containing solution showed clear signs of electroless deposition (Figures S1 and S2).

### 3.2. Cyclic Voltammetry Measurements and Characterization

CVs were performed, using solutions containing the metal salts, to investigate the presence of any faradic processes associated with reduction reactions that could be attributable to the electrodeposition of the metals. The measurements were carried out by first performing cyclovoltammetries with the blank solution (0.1 M H_2_SO_4_) only, so as to have a term of comparison between the CV concerning the metal solution and the CV of the supporting electrolyte, both carried out in the chosen range. The potential range selected was −0.6 V to 0 V vs. Ag/AgCl/sat. KCl to avoid hydrogen evolution at the low potential end and oxidation of the silicon surface at more positive potentials. For each session of cyclovoltammetry measurements with a given metal solution, three CVs were performed with the metal solution, reciprocating the solution within the electrochemical cell after each CV scan. Finally, the electrochemical cell was washed several times with the washing solution, i.e., the supporting electrolyte.

**Figure 2:**
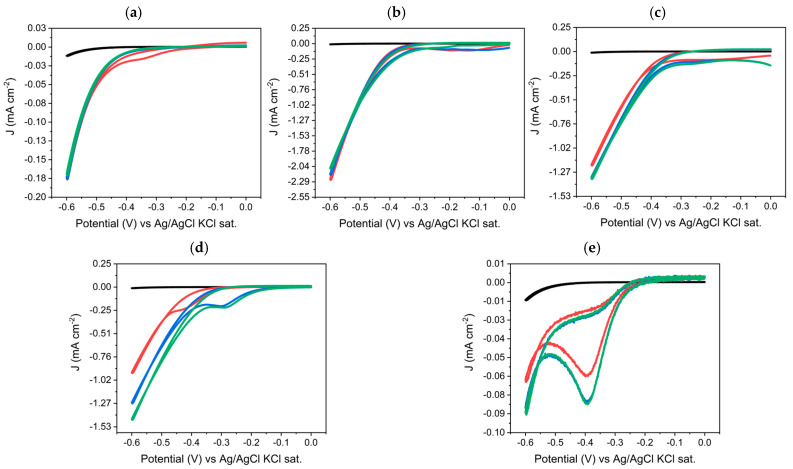
CVs performed in the potential range of −0.6 V to 0 V vs. Ag/AgCl/KCl (sat.), 10 mV/s scan rate, H_2_SO_4_ 0.1 M solution (black scans), H_2_SO_4_ 0.1 M, 1 mM metal solution, first metal solution scan (red), second metal solution scan (blue), and the third metal solution scans (green) relative to (**a**) Ni (NiSO_4_·6H_2_O); (**b**) Pd ([Pd(NH_3_)_4_]SO_4_); (**c**) Pt (K_2_PtCl_4_); (**d**) Rh (RhCl_3_·xH_2_O); and (**e**) Ru ([Ru(NH_3_)_6_]Cl_3_).

The CVs obtained for each solution are given in Figure 2. CVs were performed in the potential range of −0.6 V to 0 V vs. Ag/AgCl/KCl (sat.), with a 10 mV/s scan rate, H_2_SO_4_ 0.1 M solution (black scans), H_2_SO_4_ 0.1 M, 1 mM metal solution, the first metal solution scan (red), the second metal solution scan (blue), and the third metal solution scans (green) relative to (a) Ni (NiSO_4_·6H_2_O); (b) Pd ([Pd(NH_3_)_4_]SO_4_); (c) Pt (K_2_PtCl_4_); (d) Rh (RhCl_3_·xH_2_O); and (e) Ru ([Ru(NH_3_)_6_]Cl_3_) (Figure 2). In the first scan (red curve) of the CVs of the solution containing nickel (Figure 2a), it is possible to observe an onset of a weak and broad peak covered by the hydrogen evolution, which is not present in the second and third CVs (blue and green curves). The hypothesis could be of a possible UPD. This would suggest a particular affinity of nickel toward the substrate and thus its deposition at a less negative potential than the reduction potential predicted by the Nernst equation [58]. Subsequent depositions hypothetically would be metal on metal and consequently at more negative potentials, which is perhaps why no cathode peaks are observed in the other two CV scans.

In the CVs of palladium solution (Figure 2b) for the first two scans (blue and red curves), we observe very similar trends, while for the last CV (green), the trend is different, probably a symptom of a change in the electrode surface. In the first two CVs, we observe two cathodic peaks very close to each other, while in the last one, only a rather broadened peak that is covered by the onset of hydrogen evolution. Moreover, cathodic currents already begin to develop at 0 V. The observed cathodic currents are very high since the deposited palladium is an efficient catalyst for hydrogen evolution [59].

In the CVs of the platinum salt solution (Figure 2c), rather high cathodic currents are observed already at a potential of 0 V. This is probably due to the fact that the reduction in Pt (II) to Pt occurs already at 0 V. In the second and third CVs (blue and green), a shift in the curve to more positive potential is observed, probably due to a change in the surface area and thus a hypothetical metal deposition. The observed cathodic currents are very high since the deposited platinum is a well-known catalyst for hydrogen evolution [60].

The rhodium solution (Figure 2d) first scan shows the onset of a rather broadened peak, attributable to the reduction in the metal, which at lower potentials is covered by the cathodic current due to the evolution of hydrogen. Subsequent scans show a broadened peak again attributable to metal reduction; also, a shift in the cathodic peak to more positive potential is noted, probably due to metal deposition. The observed cathodic currents are very high, as in the previous cases; rhodium is a catalyst for the evolution of hydrogen [61].

The CVs of the ruthenium solution (Figure 2e) (red, blue, and green curves) all show a reduction peak centered at a potential of −0.4 V attributed to the reduction in Ru(III) to Ru(II) [62].

The CVs of the metal solution containing cobalt and manganese (Figure S3) show a similar trend to the scan performed with the supporting electrolyte, so it is assumed the exclusion of cathodic processes is attributable to a reduction in the metal ion.

No anodic peaks related to silicon oxidation are observed in the CVs in Figure 2, which is probably related to the value of the metal–silicon Schottky barrier; a higher potential may be needed to overcome the Schottky barrier, resulting in an increased onset potential for redox reactions [56,63].

Contrary to what is typically observed in the literature [64], no crossover (e.g., no overpotential deposition) was observed in the CVs, confirming the high affinity of the metals with silicon.

**Figure 3:**
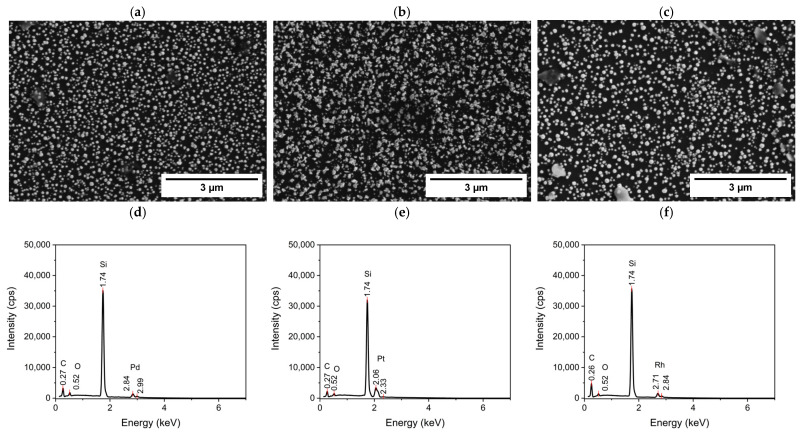
SE-SEM images and EDS-SEM spectra of the Si working electrode after the cyclovoltammetry measurements in H_2_SO_4_ 0.1 M, 1 mM metal solution relative to (**a**,**d**) 1 mM [Pd(NH_3_)_4_]SO_4_ solution; (**b**,**e**) 1 mM K_2_PtCl_4_ solution; (**c**,**f**) 1 mM RhCl_3_ solution.

The silicon electrodes used during the cyclic voltammetry measurements were analyzed by SEM-EDS in order to evaluate the morphology and composition of the surface and consequently to assess the presence of a metal deposit. SEM analysis conducted on electrodes used in CVs showed the presence of a metallic deposit for Pd, Pt, and Rh; this is confirmed by both morphological and compositional characterization (Figure 3).

The observed particles are within the nanometer size range. One possible explanation for the variation in particle sizes between the three metals is the difference in nucleation overpotentials among palladium, platinum, and rhodium, which results in varying numbers of nucleation sites. Additionally, differences in surface energy between the three metals may also contribute to the differences in particle size.

These results agree with the cathodic peaks present in the CVs performed using the solutions of these metals. In the EDS spectra, in addition to the peaks relating to the metals, which can be clearly observed for Pd, Pt, and Rh, the peaks of the silicon substrate, carbon, and oxygen due to the air contamination are also recognizable. As expected, the intensity of the peaks corresponding to the metals is relatively low due to the little amount of the deposit.

In contrast, no metal was detected for Mn, Co, Ni, and Ru (Figure S4). The SEM-EDS results of cobalt and manganese agree with what was observed in the CVs. For ruthenium, the results are consistent if we consider the cathodic peaks as the reduction in Ru (III) to Ru (II) (Figure 2e). In the case of nickel, a cathodic peak was observed in the CVs, but it did not give evidence of the metal deposition by SEM-EDS.

### 3.3. Charge-Controlled Deposition

Charge-controlled deposition of Ni, Ru, Pd, Rh, and Pt was attempted to deposit the equivalent amount of a single monolayer [16]. The theoretical deposition charge was calculated using Faraday’s laws, considering the silicon lattice constant of 543.09 pm [65], its face-centered cubic (FCC) crystal structure [20], and the 100 exposed face. The moles required to coat the substrate with a monolayer have been calculated, considering the stoichiometric silicon-metal combination that is most thermodynamically stable. The most stable phases formed by silicon and the deposited metal were identified using “the Open Quantum Materials Database” (OQMD) [47]. The calculated charge was increased by 15% to consider capacitive contributions. The properties of the metals, the estimated charges, and deposition potentials derived previously from the peaks present in the CVs are summarized in Table 1.

**Figure 4:**
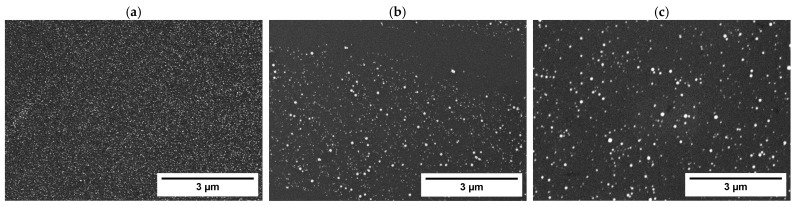
SE-SEM images of the deposit obtained through charge-controlled deposition on the Si working electrode in H_2_SO_4_ 0.1 M, 1 mM metal solution (**a**) 1 mM [Pd(NH_3_)_4_]SO_4_ solution; (**b**) 1 mM K_2_PtCl_4_ solution; (**c**) 1 mM RhCl_3_ solution.

The SEM analysis (Figure 4) of the samples showed the deposition of particles in the nanometer size range for palladium, platinum, and rhodium. Notably, obtaining metal nanoparticles via electrodeposition on silicon is significant for several reasons, such as enhancing catalytic properties, electrical conductivity, and magnetic properties, while leveraging all the advantages of electrochemical deposition (e.g., tunable properties of the deposit, scalability, and low-cost fabrication). However, in the case of charge-controlled deposition of nickel and ruthenium, no detectable presence of these metals was detected. Although no Ni and Ru deposits electrodeposited on n-silicon were obtained under the conditions used, such results are reported in the literature and were obtained using different working conditions than those used for this article. These conditions were not ideal for the implementation with the experimental setup used in this work [66] or were not suitable for obtaining deposits with the characteristics sought [67].

**Figure 5:**
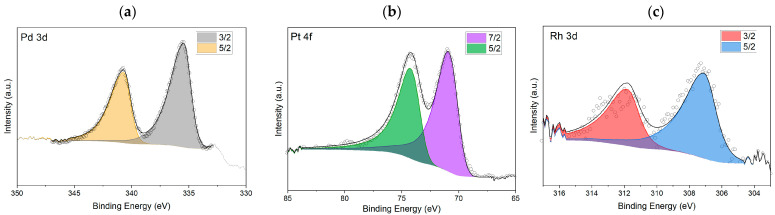
XPS analysis performed on the silicon electrodes used after charge-controlled deposition on the Si working electrode in H_2_SO_4_ 0.1 M, and 1 mM metal solution: (**a**) 1 mM [Pd(NH_3_)_4_]SO_4_ solution; (**b**) 1 mM K_2_PtCl_4_ solution; (**c**) 1 mM RhCl_3_ solution.

XPS analyses were performed on the samples to confirm the presence of the metals and their oxidation state (Figure 5). The presence of the peaks related to the photoelectronic emission of Pd, Rh, and Pt was confirmed by studying the corresponding emission lines. In Figure 5a, the doublet constituting the 3D emission of palladium is analyzed. The fitting of the doublet was performed as per the literature, considering a Shirley-type background, an LA (α = 1.9, β = 7, m = 2) curve shape [44,68], a ratio of the d3/2 and d5/2 peak areas of 0.667, the same full width at half maximum (FWHM) for the 2 peaks, and the relative binding energies of the 2 peaks of 5.26 eV. A value of 335.4 eV was obtained from the peak analysis, which, according to the literature, is within the expected value for metallic palladium (335.4 eV).

In Figure 5b, the doublet constituting the 3f emission of platinum is analyzed. The doublet was fitted as per the literature [69], considering a Shirley-type background, an LA(1.2, 85, 70) curve shape, a ratio between the f5/2 and 7/2 peak areas of 0.75, the same FWHM for the 2 peaks, and the relative binding energies of the 2 peaks (3.33 eV).

A value of 71.0 eV was obtained from the analysis of the peak, which, according to the literature, is within the expected value for metallic platinum (71.1 eV).

In Figure 5c, the doublet constituting the 3D emission of rhodium is analyzed. The doublet was fitted as per the literature [70], considering a Shirley-type background, an LA(1.2, 3, 2) curve shape, a ratio between the d3/2 and d5/2 peak areas of 0.667, the same FWHM for the 2 peaks, and the relative binding energies of the 2 peaks (4.74 eV). A value of 307.2 eV was obtained from the peak analysis, close to the value of binding energy present in the literature for rhodium metal (307.0 eV).

XPS analysis showed no metal presence in the case of nickel and ruthenium charge-controlled deposition, confirming the EDS-SEM analyses results.

### 3.4. Multi-Cycle Charge-Controlled Deposition

Multi-cycle charge-controlled depositions were performed to evaluate the possibility of obtaining a deposit whose morphology can be modulated as deposition cycles change, varying between a thin film and a nanostructured deposit. The metal selected for these tests was rhodium. This metal was found to be the most promising for these experiments, as it exhibits a cathodic peak attributable to the deposition of the metal within the selected potential range and does not exhibit deposition phenomena occurring in an electroless mode. To the best of our knowledge, there are limited articles in the literature dealing with the rhodium deposition on silicon.

**Figure 6:**
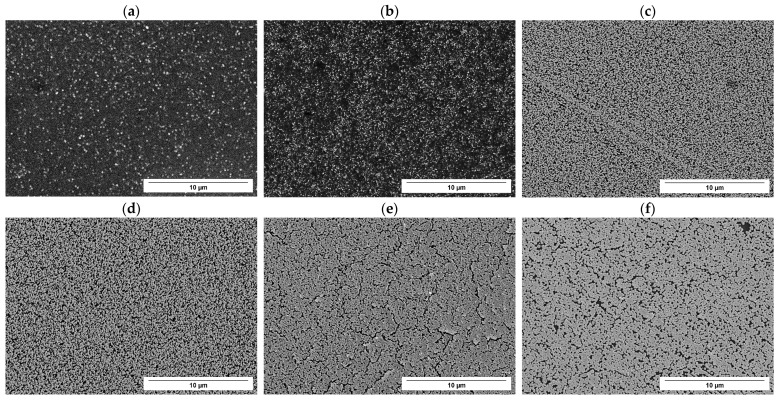
SEM images of the deposits obtained through a variable number of charge-controlled deposition cycles on the Si working electrode in H_2_SO_4_ 0.1 M and RhCl_3_ 1 mM solution: (**a**) 1 cycle; (**b**) 10 cycles; (**c**) 20 cycles; (**d**) 30 cycles; (**e**) 40 cycles; and (**f**) 50 cycles.

To observe the variation in deposit morphology as the number of deposition cycles changes, five charge-controlled deposition sessions were carried out at 1 cycle, 10 cycles, 20 cycles, 30 cycles, 40 cycles, and 50 cycles, respectively. Where one cycle corresponds to 256 + 15% µC (according to Table 1). In Figure 6, it is possible to observe an increasing coverage of rhodium on the silicon surface; however, even at 50 cycles, there is not a total coverage of the surface, and a complete film is not observed. Instead, a nanostructured porous deposit of rhodium was observed.

**Figure 7:**
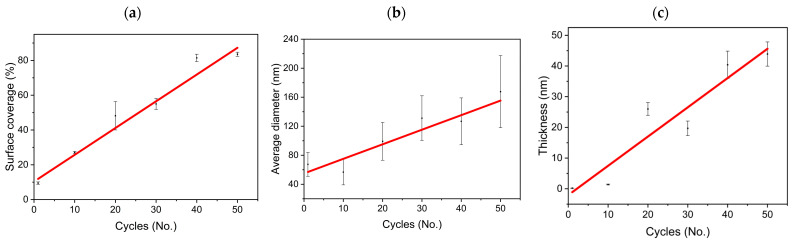
(**a**) Surface coverage trend; (**b**) average particle diameter size trend; (**c**) equivalent thickness trend of the deposits, obtained through a variable number of charge-controlled deposition cycles (1, 10, 20, 30, 40, and 50 cycles) on the Si working electrode using H_2_SO_4_ 0.1 M and RhCl_3_ 1 mM solutions.

Quantification of silicon surface coverage was performed using the ImageJ (Version 1.54d) program by analyzing SEM images related to controlled rhodium deposition at 1 cycle, 10 cycles, 20 cycles, 30 cycles, 40 cycles, and 50 cycles (Table S2). Figure 7a shows the overall increasing trend of the surface coverage as the number of deposition cycles rises. For a charge-controlled deposition at 50 cycles, a surface coverage of almost 85 percent is obtained.

Rhodium particle size distribution analyses were also performed using the ImageJ program. In Figure 7b the average particle diameter is shown, and an overall increasing trend is visible. The results show an average diameter of about 67 nm for one-cycle deposition and up to about 168 nm for 50-cycle deposition, confirming the achievement of a nanostructured deposit.

EDS measurements were carried out using the Layer Probe program of AZteclive. These measurements were aimed at investigating the average equivalent thickness of the metallic rhodium deposit on the silicon surface. In addition, to improve the fitting of the EDS spectrum, an additional layer containing carbon and oxygen in varying amounts was included within the theoretical layered model; this allows for oxides and carbon residues found on the surface to be considered.

Figure S5 presents the EDS spectra of the sample and the values of thickness at 1 cycle, 10 cycles, 20 cycles, 30 cycles, 40 cycles, and 50 cycles. The analysis yielded the average metal deposit thickness for each cycle. The thickness obtained for each deposit was plotted as a function of the number of cycles in Figure 7c.

The observed trend aligns with previous results regarding surface coverage and the average particle diameter. The data demonstrate that we have achieved an average thickness of less than a few hundred nanometers. Consequently, we can confirm that a nanostructured deposition has been successfully obtained [71].

## 4. Conclusions

In this work, we evaluated the controlled electrochemical deposition of metals on silicon surfaces, exploring the potential for underpotential deposition (UPD), thin and ultra-thin films, and nanostructured deposits. Mn, Co, Ni, Ru, Pd, Rh, and Pt were selected based on their high affinity for silicon, as predicted by DFT calculations. Electrochemical characterization revealed cathodic peaks for Ni, Pd, Ru, Rh, and Pt, with further SEM-EDS and XPS analyses confirming metallic deposits only for Pd, Rh, and Pt, forming nanosized particles. Despite the theoretically high affinity of the chosen metals with silicon, UPD phenomena were not observed for any of the studied metals under the experimental conditions.

The study of rhodium electrodeposition was deepened: multi-cycle charge-controlled deposition on n-doped silicon demonstrated tunable surface coverage and particle size, starting from an average diameter of 67 nm for 1 cycle and reaching 168 nm after 50 deposition cycles, with a surface coverage of 84%. The equivalent deposit thickness, estimated in tens of nanometers, correlated with surface coverage and particle size, confirming the formation of nanostructured rhodium. These results highlight the potential of rhodium electrodeposition on silicon to develop materials with tailored structural, electrical, and catalytic properties. Future work should focus on optimizing deposition processes and assessing the long-term stability and adhesion of these nanostructures to expand their applicability in catalysis, electronics, and sensing.

## Figures and Tables

**Table 1:** Experimental parameters for charge-controlled deposition of Rh, Pt, Pd, Ru, and Ni.

	Oxidation Number of the Metal in the Salt	Metal/Silicon Most Stable Stoichiometry	Number of Electrons Exchanged	Charge (µC)	Peak Potential
Rh	3+	1:1	3	256 + 15%	−0.425 V
Pt	2+	1:2	4	341 + 15%	−0.150 V
Pd	2+	1:2	4	341 + 15%	−0.170 V
Ru	3+	1:1	3	256 + 15%	−0.400 V
Ni	2+	1:2	4	341 + 15%	−0.375 V

## Data Availability

The original contributions presented in the study are included in the article and Supplementary Materials; further inquiries can be directed to the corresponding authors.

## Supplementary Materials

The following supporting information can be downloaded at https://www.mdpi.com/article/10.3390/nano14242042/s1, Electroless deposition; Table S1: Radio Corporation of America (RCA) procedure used for the treatment of the silicon wafer; Table S2: Surface coverage values of rhodium deposits on silicon obtained through a variable number of charge-controlled deposition cycles on the Si working electrode in H_2_SO_4_ 0.1 M and RhCl_3_ 1 mM solution, acquired by processing SE-SEM images using ImageJ software; Figure S1: SEM image of the deposit obtained through electroless deposition on Si working electrode in H_2_SO_4_ 0.1 M, K_2_PtCl_4_ 1 mM solution; Figure S2: EDS-SEM spectra of the Si working electrode used for the cyclovoltammetry measurements using a H_2_SO_4_ 0.1 M, 1 mM K_2_PtCl_4_ solution; Figure S3: CVs performed in the potential range of −0.6 V to 0 V, vs. Ag/AgCl/KCl (sat.), 10 mV/s scan rate, of H_2_SO_4_ 0.1 M solution (black scans), H_2_SO_4_ 0.1 M, 1 mM metal solution, first metal solution scan (red), second metal solution scan (blue), third metal solution scans (green), relative to (a) CoSO_4_·7H_2_O; (b) MnSO_4_·H_2_O; Figure S4: EDS-SEM spectra of the Si working electrode used for the cyclovoltammetry measurements using H_2_SO_4_ 0.1 M, 1 mM metal solution (a) CoSO_4_·7H_2_O; (b) MnSO_4_·H_2_O; (c) NiSO_4_·6H_2_O; (d) [Ru(NH_3_)_6_]Cl_3_; Figure S5: EDS-SEM spectra of the deposits obtained through a variable number of charge-controlled deposition cycles on the Si working electrode in H_2_SO_4_ 0.1 M and RhCl_3_ 1 mM solution: (a) 1 cycle; (b) 10 cycles; (c) 20 cycles; (d) 30 cycles; (e) 40 cycles; and (f) 50 cycles. Reference [72] are cited in the supplementary materials.
