# Supplementary material for: Electrodeposition of Nanostructured Metals on n-Silicon and Insights into Rhodium Deposition
Source: Nanomaterials (Basel). 2024 Dec 20;14(24):2042. doi: 10.3390/nano14242042 (PMC11679951; doi:10.3390/nano14242042)
Supplement: Supplementary file 1 [file nanomaterials-14-02042-s001.zip › nanomaterials-3341499-supplementary.pdf]

nanomaterials

SUPPLEMENTARY MATERIAL OF

Electrodeposition of Nanostructured Metals on n-Silicon and Insights into Rhodium Deposition.

Giulio Pappaianni[1], Francesco Montanari[1], Marco Bonechi[1,2], Giovanni Zangari[3], Walter Giurlani[1,2,*] and Massimo Innocenti[1,2,*]

[1] Dipartimento di Chimica "Ugo Schiff", Università degli Studi di Firenze, via della Lastruccia 3, 50019 Sesto Fiorentino, Italy; giulio.pappaianni@unifi.it (G.P); francesco.montanari@unfi.it (F.M); marco.bonechi@unifi.it (M.C.)
[2] Consorzio Interuniversitario Nazionale per la Scienza e Tecnologia dei Materiali (INSTM), via G. Giusti 9, 50121 Florence, Italy; walter.giurlani@unifi.it (W.G.); m.innocenti@unifi.it (M.I.)
[3] Department of Materials Science and Engineering, University of Virginia, Charlottesville, 22904, VA, United States; gz3e@virginia.edu (G.Z.)
* Correspondence: walter.giurlani@unifi.it and m.innocenti@unifi.it

Citation: To be added by editorial staff during production.

Academic Editor: Firstname Lastname

Received: date
Revised: date
Accepted: date
Published: date

Copyright: © 2023 by the authors. Submitted for possible open access publication under the terms and conditions of the Creative Commons Attribution (CC BY) license (https://creativecommons.org/licenses/by/4.0/).

Electroless deposition

The purpose of the electroless deposition was to investigate the possibility of obtaining a metal deposit without the aid of an external current applied to the working electrode. The deposition was performed by immersing the silicon electrode inside the appropriate metal solution (1 mM metal salt, H_2SO_4 0.1 M, deaerated for 30 minutes before the deposition and kept in a nitrogen atmosphere during the experiment), the deposition lasted for a total time of 300 seconds. The test was performed on solutions containing Pd, Pt, Rh, Ru, and Ni, respectively.

SEM measurements were performed to assess the presence of a deposit and its morphology. Electroless deposition was obtained under these conditions only for the solution containing platinum.

The surface of the electrode used in electroless platinum deposition can be observed in **Figure S1**, showing a not-homogeneous deposit, with the presence of particles with sub-micron size both isolated and aggregated. This was especially noted in scratches present on the electrode surface. A symptom that nucleation and particle growth occur preferentially along the imperfections that constitute the surface. EDS measurements confirmed the presence of the platinum deposit by compositional analysis of the electrode surface (**Figure S2**). Several articles in the literature [1] confirm the possibility of electroless deposition of platinum on a silicon surface.

Table S1. Radio Corporation of America (RCA) procedure used for the treatment of the silicon wafer.

Step	Process	Duration
1	H_2SO_4-H_2O_2 (4:1)	5 minutes
2	milliQ water sonication	5 minutes
3	H_2SO_4-H_2O_2 (4:1)	5 minutes
4	milliQ water sonication	5 minutes

5	H_2SO_4-H_2O_2 (4:1)	5 minutes
6	milliQ water sonication	5 minutes
7	H_2SO_4-H_2O_2 (4:1)	5 minuti
8	milliQ water sonication	5 minutes
9	NH_4OH-H_2O_2-H_2O (0.05:1:5), 90 °C	10 minutes
10	milliQ water flushing	Few seconds
11	AcOH	10 minutes
12	EtOH	10 minutes
13	milliQ water flushing	Few seconds
14	HCl-H_2O_2-H_2O (1:1:6), 90 °C	10 minutes
15	milliQ water flushing	Few seconds
16	HF 2 M	10 minutes
17	milliQ water flushing	Few seconds

Table S2. Surface coverage values of rhodium deposits on silicon obtained through a variable number of charge controlled deposition cycles on Si working electrode in H_2SO_4 0.1 M, $RhCl_3$ 1mM solution, acquired by processing SE-SEM images using ImageJ software.

**Cycles (No.)**	**Surface coverage (%)**			**Average (%)**	**Error (±)**
1	7.74	10.30	10.30	9.45	0.70
10	26.07	27.12	28.18	27.12	0.50
20	68.17	37.31	39.07	48.18	8.17
30	49.99	62.62	52.33	54.98	3.17
40	78.56	79.09	86.66	81.44	2.14
50	86.44	82.33	81.88	83.55	1.18

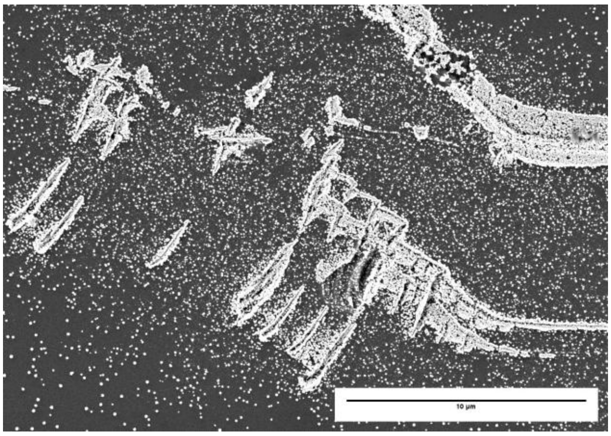

Figure S1. SEM image of the deposit obtained through electroless deposition on Si working electrode in H_2SO_4 0.1 M, K_2PtCl_4 1mM solution.

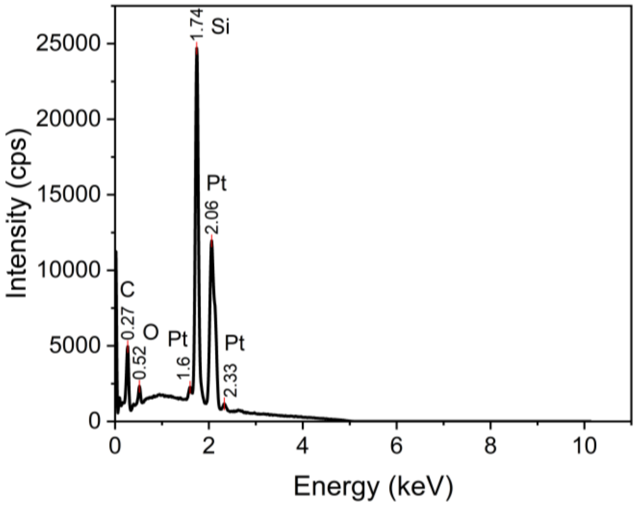

Figure S2. EDS-SEM spectra of the deposit obtained through electroless deposition on Si working electrode in H_2SO_4 0.1 M, K_2PtCl_4 1mM solution.

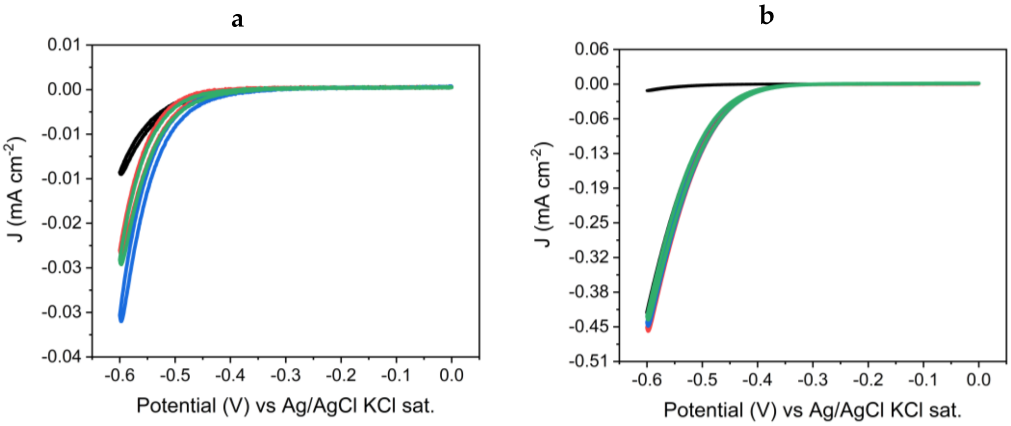

Figure S3. CVs performed in the potential range of -0.6 V to 0 V, vs Ag/AgCl/KCl (sat.), 10 mV/s scan rate, of H2SO4 0.1 M solution (black scans), H2SO4 0.1 M, 1 mM metal solution, first metal solution scan (red), second metal solution scan (blue), third metal solution scans (green), relative to a) CoSO4·7H2O; b) MnSO4·H2O.

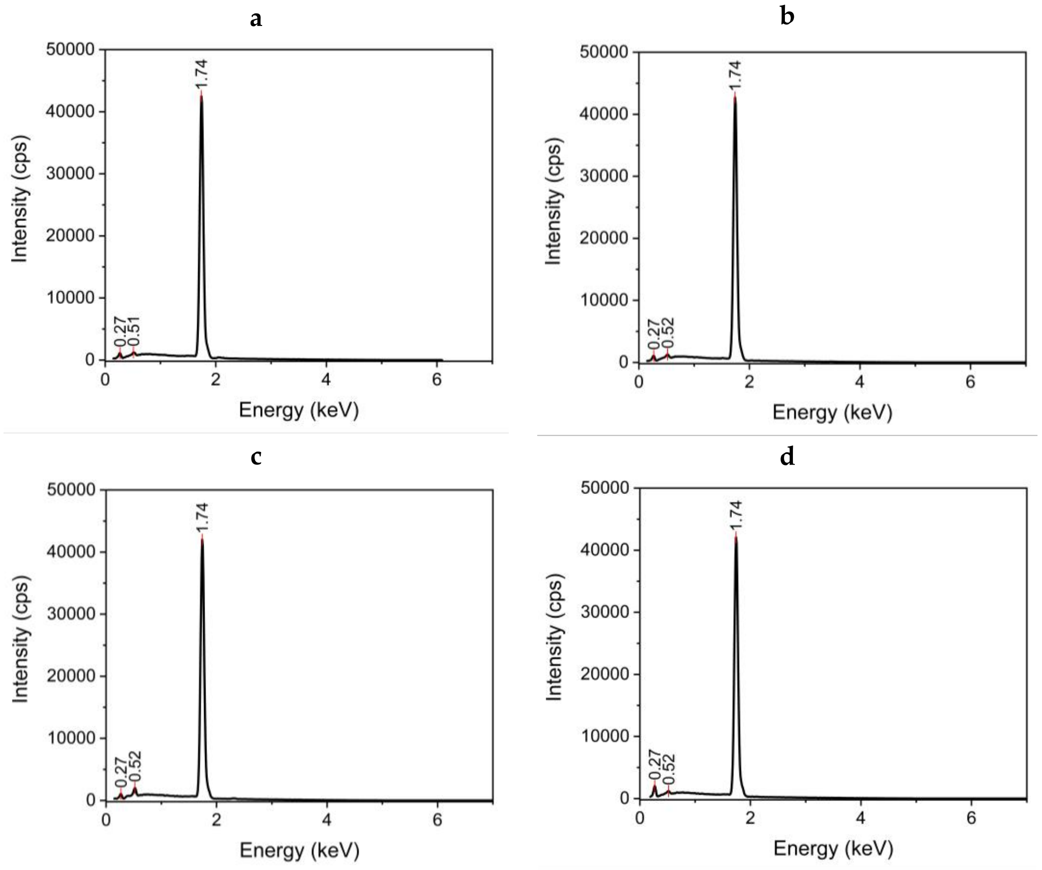

Figure S4. EDS-SEM spectra of the Si working electrode used for the cyclovoltammetry measurements using H_2SO_4 0.1 M, 1 mM metal solution a) $CoSO_4{\cdot}7H_2O$; b) $MnSO_4{\cdot}H_2O$; c) $NiSO_4{\cdot}6H_2O$; d) $[Ru(NH_3)_6]Cl_3$.

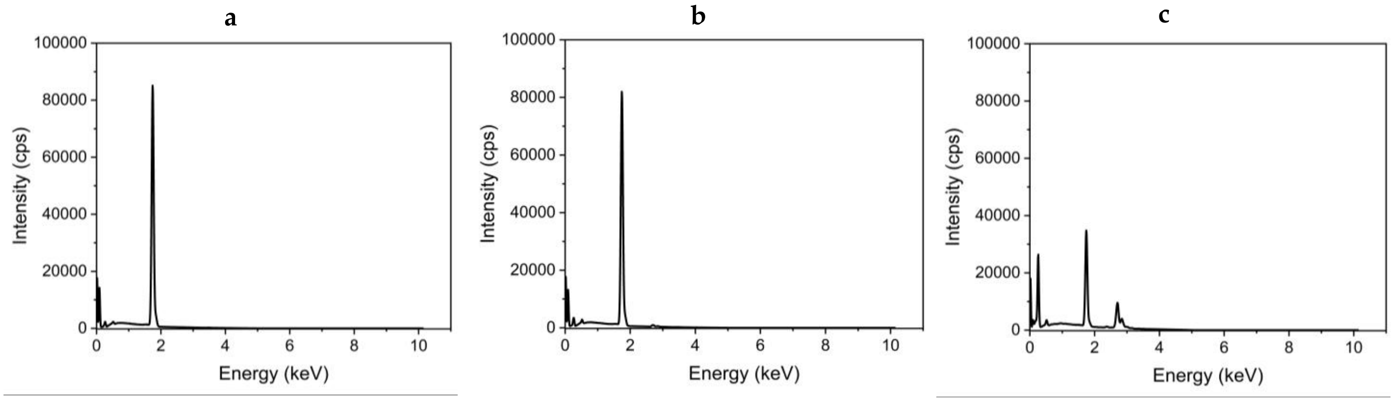

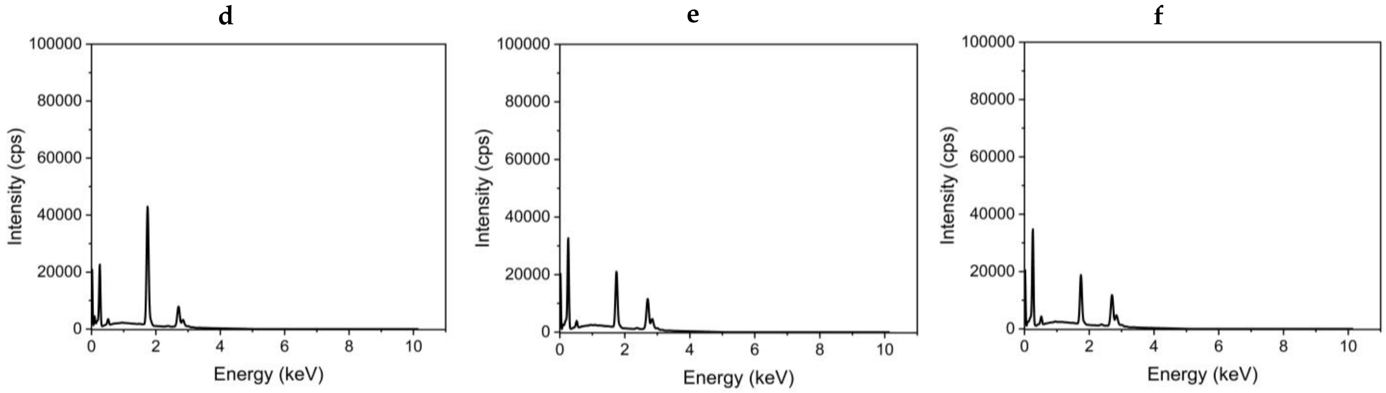

Figure S5. EDS-SEM spectra of the deposits obtained through a variable number of charge controlled deposition cycles on Si working electrode in H_2SO_4 0.1 M, $RhCl_3$ 1mM solution: a) 1 cycle; b) 10 cycles; c) 20 cycles; d) 30 cycles; e) 40 cycles; and f) 50 cycles.

References

1. Kuznetsov, G. V.; Skryshevsky, V.A.; Vdovenkova, T.A.; Tsyganova, A.I.; Gorostiza, P.; Sanz, F. Platinum Electroless Deposition on Silicon from Hydrogen Fluoride Solutions: Electrical Properties. *J. Electrochem. Soc.* **2001**, *148*, C528, doi:10.1149/1.1382591.
